# Coaching and Individualized Learning in Competency‐Based Medical Education: Framework Development and Research Priorities

**DOI:** 10.1002/aet2.70148

**Published:** 2026-04-14

**Authors:** Simanjit K. Mand, Meg Wolff, Nicole Zamignani, Sarah R. Williams, Sally A. Santen, Kevin R. Scott, Alexander Garrett, Charles Brown, Jung G. Kim, Michele L. Dorfsman, Holly Caretta‐Weyer, Jeremy Branzetti

**Affiliations:** ^1^ Emergency Medicine University of Wisconsin School of Medicine and Public Health Madison Wisconsin USA; ^2^ Emergency Medicine and Pediatrics University of Michigan Medical School Ann Arbor Michigan USA; ^3^ Emergency Medicine Stanford University School of Medicine Stanford California USA; ^4^ The Coaching Office: Advancing Coaching in Healthcare and Medical Education (COACHME@Stanford) Stanford USA; ^5^ Stanford University School of Medicine Stanford California USA; ^6^ Emergency Medicine and Medical Education University of Cincinnati School of Medicine Cincinnati Ohio USA; ^7^ Emergency Medicine Geisinger Commonwealth School of Medicine Scranton Pennsylvania USA; ^8^ Emergency Medicine University of Washington School of Medicine Seattle Washington USA; ^9^ Emergency Medicine Oregon Health and Science University School of Medicine Portland Oregon USA; ^10^ Emergency Medicine New York University Grossman School of Medicine New York City New York USA; ^11^ Emergency Medicine University of Pittsburgh School of Medicine Pittsburgh Pennsylvania USA; ^12^ Emergency Medicine, Associate Dean, Admissions and Assessment Stanford University School of Medicine Palo Alto California USA; ^13^ Emergency Medicine Yale University School of Medicine New Haven Connecticut USA

## Abstract

**Introduction:**

Competency‐based medical education (CBME) is a learner‐centric and outcomes‐based approach to medical training that is essential to improve patient care. Coaching and individualized learning have been proposed to catalyze a core component of CBME, “competency‐focused instruction”. While coaching and individualized learning hold great theoretical and conceptual benefit to drive future educational initiatives, there remains a paucity of literature on how to incorporate them successfully, particularly in graduate medical education (GME) in Emergency Medicine (EM). A national workgroup of academic EM faculty with expertise in these areas was assembled to identify priority research questions that address key knowledge gaps in the design, delivery, and outcomes of coaching and individualized learning within a CBME framework.

**Methods:**

Twelve members with diverse representation convened to conduct a three‐round modified Delphi process to help identify high‐impact scholarly questions that would inform a discipline‐specific research agenda within the broader 2025 Society for Academic Emergency Medicine (SAEM) Consensus Conference on CBME.

**Results:**

The final 10 research questions identify a strategic direction to focus future scholarly initiatives relevant to EM and coaching in CBME. Four question categories were identified: coaching effectiveness and outcomes, coaching program design, faculty development and coaching competencies, and learner‐centered factors.

**Implications:**

The consensus‐building process revealed a pivotal period for EM's transition to CBME, with the need for more rigorous, evidence‐driven understanding of coaching and individualized learning mechanisms, outcomes, and best practices to optimally prepare faculty and learners for successful implementation. The potential impact of this work not only affects individual learners but brings forth considerations for EM faculty, training programs, and national stakeholders to inform a supportive infrastructure and relevant resources to successfully actualize CBME in EM.

## 1 Introduction

### 1.1 CBME: What Is It and Why Do We Need It?

Competency‐based medical education (CBME) is a learner‐centered, outcomes‐driven framework that emphasizes the achievement of competency rather than completion of a time‐based curriculum [1]. This approach acknowledges that learners progress at variable rates and may need tailored training times to be adequately prepared for future independent practice. Competency achievement is tracked over time using direct observations of clinical performance as the foundation for formative and summative assessment [2, 3]. When combined with informed self‐assessment and a growth mindset, this approach ideally leads to the development of self‐regulated, lifelong learners prepared for evolving clinical demands.

CBME has been prioritized by the Accreditation Council for Graduate Medical Education (ACGME) as a key initiative in modernizing graduate medical education (GME) to address two intertwining, fundamental needs: (1) to ensure our trainees have achieved competency to safely practice independently, and (2) to better serve patient and societal needs [4]. However, within the current medical education structure, leadership teams in both medical schools and residency programs have reported graduating trainees they would not trust to take care of their own family members [5, 6]. Thus, more rigorous methods of ensuring learner competency in all domains (communication skills, professionalism, medical knowledge, clinical practice, etc.) are critical to more safely progressing trainees. This calls for a collaborative, specialty‐wide effort to better standardize the harnessing and use of learner data to inform a tailored approach to longitudinal assessment and tracking for each individual trainee.

The overarching goal of CBME's reimagination of medical training is to offer a new approach and paradigm to optimize patient care [7]. Healthcare systems should ideally improve population health, provide equitable care, and enhance patients' care experiences while simultaneously promoting workforce wellbeing [7]. However, our current system consistently underperforms in patient outcomes [8], satisfaction [9], cost containment [10], and provider wellness [11]. While many of these challenges are complex, rethinking how we train future physicians is a key consideration in building towards an ideal state. CBME seeks to ensure learners have demonstrated competency not only in medical knowledge, but also in addressing the needs of patients, communities, and healthcare systems [12], preparing them to thrive in an ever‐changing clinical landscape.

### 1.2 Coaching and Individualized Learning in Competency‐Focused Instruction

One of CBME's five core components is “competency‐focused instruction,” which emphasizes individualized teaching based on each learner's developmental stage and pace [13]. This requires direct observation of a learner's performance, provision of formative teaching and feedback, identification of knowledge and/or performance gaps, creation of a plan to address gaps, and tracking of progress towards achieved competency over time [12]. As learners have historically struggled with accurately self‐assessing their performance [14, 15], identifying areas to focus on in support of their growth [16], and creating meaningful and achievable goals independently [17], they would benefit from external support to help with this process.

Coaching in medical education is becoming increasingly recognized as a valuable strategy for learners within the CBME framework, particularly around competency‐focused training. Whether delivered in‐the‐moment or through longitudinal relationships [18], coaching employs intentional strategies to engage and empower learners to reach their full potential [19]. Coaching in medical education supports learners in self‐assessing their performance, identifying gaps between current abilities and desired competencies, clarifying and co‐creating specific and feasible goals, and taking ownership of their growth, all within a supportive and collaborative partnership [20, 21, 22]. By creating a psychologically safe space for reflection and feedback, coaching fosters a growth mindset, supports intentional self‐development, and has the potential to meaningfully impact both educational outcomes and the quality of healthcare delivery [23]. With its focus on real‐world performance and self‐regulated learning, coaching is well‐suited to support the development of the learner in a CBME framework.

Coaching is a discreet skillset separate from other common faculty roles of teaching, advising, and mentoring, and requires dedicated training for medical educators who will serve as coaches in the CBME model [24]. Understanding coaching theories and the influence of positive psychology can inform the foundational approach to coaching in medical education [25]. Understanding and practicing relational and conversational skills (building a relationship of trust with minimized hierarchy, probing self‐reflection/assessments without judgment, encouraging learner self‐awareness, active listening) can help transition a focus from the faculty being the expert in the interaction to a more learner‐focused session. Helping a learner navigate emotions and recognize the impact on performance can assist in developing self‐regulatory behaviors to help in future scenarios. Developing coaching skills (guiding critical thinking and metacognition, cultivating learner well‐being, improving learner self‐efficacy) can help foster the development of Master Adaptive Learners [25]. Faculty may already use some of these skills with learners in their other roles as educators; however, using these skills with intentionality and effectiveness is itself a competency that requires practice and honing.

Coaching utilizes various tools to assist learners in making progress on their goals, with individualized learning plans (ILPs) being a potentially helpful resource in medical training. Once primarily used for remediation, ILPs are now viewed as valuable tools for all learners [26]. As written documents, ILPs can help learners focus their learning on their zone of proximal development while also helping build accountability. Beyond this, ILPs can diligently track learner progress towards competency achievement. To this end, multiple organizations, including the ACGME, American Medical Association (AMA), and Association of American Medical Colleges (AAMC), recommend that all learners create ILPs. This wholesale adoption normalizes and affirms that every learner progresses along a continuum from novice to expert and reinforces the habits of self‐directed, lifelong learning. When combined with multisource performance data, thoughtful self‐reflection, and coach guidance, ILPs can help learners develop specific, actionable goals that support continuous development [27].

### 1.3 Current Gaps in Coaching for GME

As noted, coaching and individualized learning can theoretically augment the learner‐focused approach in CBME; however, like any proposed intervention to improve healthcare outcomes [28], they deserve rigorous scrutiny prior to widespread integration and implementation. Although coaching is a crucial component of successful CBME implementation in other countries [18, 29], and coaching competencies have been previously described in the literature [20, 25, 30], there are many logistical gaps in how to optimally incorporate coaching in medical education and a paucity of literature demonstrating its effectiveness in this domain. Further, the use of coaching specifically for EM training is mostly unexplored. While some individual EM residency programs have already implemented a coaching‐based strategy to assist with individualized learning of their trainees, the approaches are variable and have had mixed acceptability from both residents and faculty [31, 32]. Further research on best practices and standardization for coaching and individualized learning approaches are required to assist with EM‐wide implementation with CBME.

Thus, the objective of our working group was to identify priority research questions that address key knowledge gaps in the design, delivery, and outcomes of coaching within a CBME framework to inform a scholarly agenda for the next 10 years.

## 2 Methods

### 2.1 Study Design, Setting, and Population

We convened a nationally representative workgroup focusing on Coaching and Individualized Learning within the CBME in EM Consensus Conference at the Society for Academic Emergency Medicine (SAEM) Annual Meeting on May 16, 2025. This consensus conference was co‐sponsored by the Council of Residency Directors in EM (CORD), the American Board of Emergency Medicine (ABEM), and the Association for Academic Chairs in EM (AACEM) and supported by the AMA Reimagining Residency Initiative. Prospective committee members were identified by their content expertise in coaching, individualized learning, and CBME, and sampled from the EM GME community at large (Data S1). Twelve members were selected based on these criteria to ensure diverse faculty representation in: training program geographic location, training program format, academic rank, and faculty gender, race, and ethnicity. The workgroup was chaired by authors MW and JB, both of whom were selected for their extensive experience in both coaching and GME leadership.

The workgroup convened a monthly virtual meeting between January and May 2025. The objective was to identify high‐impact scholarly questions that would inform a national competency‐based coaching research agenda within the broader 2025 SAEM Consensus Conference on CBME.

### 2.2 Literature Review

A literature review was conducted to gather and build upon currently existing medical literature for coaching use with learners. The search was conducted using multiple databases (PubMed, Scopus, Web of Science, Google Scholar, and the Education Resources Information Center (ERIC)) and used the following search terms: (coach or coaching) AND (competency‐based medical education); (coach or coaching) AND (competency) AND (medical education). Search criteria targeted medical learners (medical students, residents, and fellows) and focused on both performance coaching (involving direct observations) and longitudinal or portfolio coaching. Articles published in the English language in the past 10 years were included (around or following the time of this selected landmark coaching publication [33]). Reviews, commentaries, editorials, and letters were excluded.

260 publications were initially identified and screened by the two workgroup chairs for relevance and appropriateness based on title and abstract, yielding 81 articles for workgroup review.

These articles were divided equally and assigned to 5 subgroup dyads within the workgroup. The subgroups were instructed to read each assigned article with a focus on identifying underexplored areas of inquiry as they apply to coaching or individualized learning within the CBME context.

### 2.3 Identifying Research Questions

Using Google Docs, workgroup members individually drafted potential research questions for scholarly inquiry that were prompted by the literature review, yielding 101 unique responses relevant to CBME coaching and individualized learning. The full group reviewed the proposed research questions for duplicates, redundancy, and relevance, yielding a total of 65 questions at the start of the Delphi process.

We then employed a three‐round modified Delphi process to iteratively refine and prioritize research questions and reach workgroup consensus, using Qualtrics (Provo, UT) to collect data. Rounds 1 and 2 were designed to function as a screening and refinement phase, with workgroup members rating each question using a 3‐point narrative scale based on the constructs of importance and relevance (“definitely include”; “possibly include”; and “definitely exclude”). Open‐ended comment fields were available for each question for workgroup members to provide reasoning behind their vote or other feedback on question wording or concept. Voting thresholds to achieve consensus were set a priori at 70% agreement for items scored as either “definitely include” or “possibly include” to proceed to the subsequent round. Questions meeting a ≥ 70% “definitely include” threshold in any round were advanced directly to the final list and removed from further voting. These thresholds were selected based on prior Delphi methodology literature demonstrating that consensus is most commonly defined using percentage agreement cutoffs in the 70%–80% range [34]. New suggested questions or revisions of prior questions (including merging questions with similar concepts) were discussed as a group and included in subsequent voting rounds.

The purpose of the third and final Delphi round differed intentionally from earlier rounds. Rather than serving as an additional quality filter, Round 3 was designed as a prioritization exercise to identify a focused and feasible set of research questions to advance to the in‐person SAEM consensus conference. In this round, remaining questions that had met prior inclusion criteria were presented, and workgroup members were asked to select up to 10 questions they believed should be prioritized for inclusion in a national research agenda. This forced‐choice approach is commonly used in Delphi‐based agenda‐setting studies to move from a broad set of acceptable items to a finite, actionable list. The target of 10 questions was selected a priori to align with the structure and time constraints of the SAEM consensus conference and to ensure that each question could be meaningfully discussed with a broader group of stakeholders. A two‐thirds majority (67%) threshold was used for inclusion in this final round, resulting in a final prioritized list of 10 research questions.

Between each round and prior to individual voting, the group convened virtually to interpret voting patterns, refine question phrasing, assess conceptual overlap, and confirm that retained items represented distinct and meaningful areas of inquiry. When questions demonstrated conceptual overlap without being fully redundant, the group engaged in structured discussion to determine whether the items reflected distinct constructs or could be meaningfully consolidated. Decisions were guided by the goal of preserving conceptual breadth while maintaining clarity, specificity, and feasibility for future empirical study.

### 2.4 Conference Agenda

At the 2025 SAEM Consensus Conference, our workgroup facilitated discussion of these final 10 questions with additional on‐site attendees for a total of 20 participating stakeholders. Discussions were conducted in two breakout sessions. The results of these sessions, including final prioritization and cross‐group synthesis, are presented in a complementary manuscript [35].

## 3 Results

**FIGURE 1:**
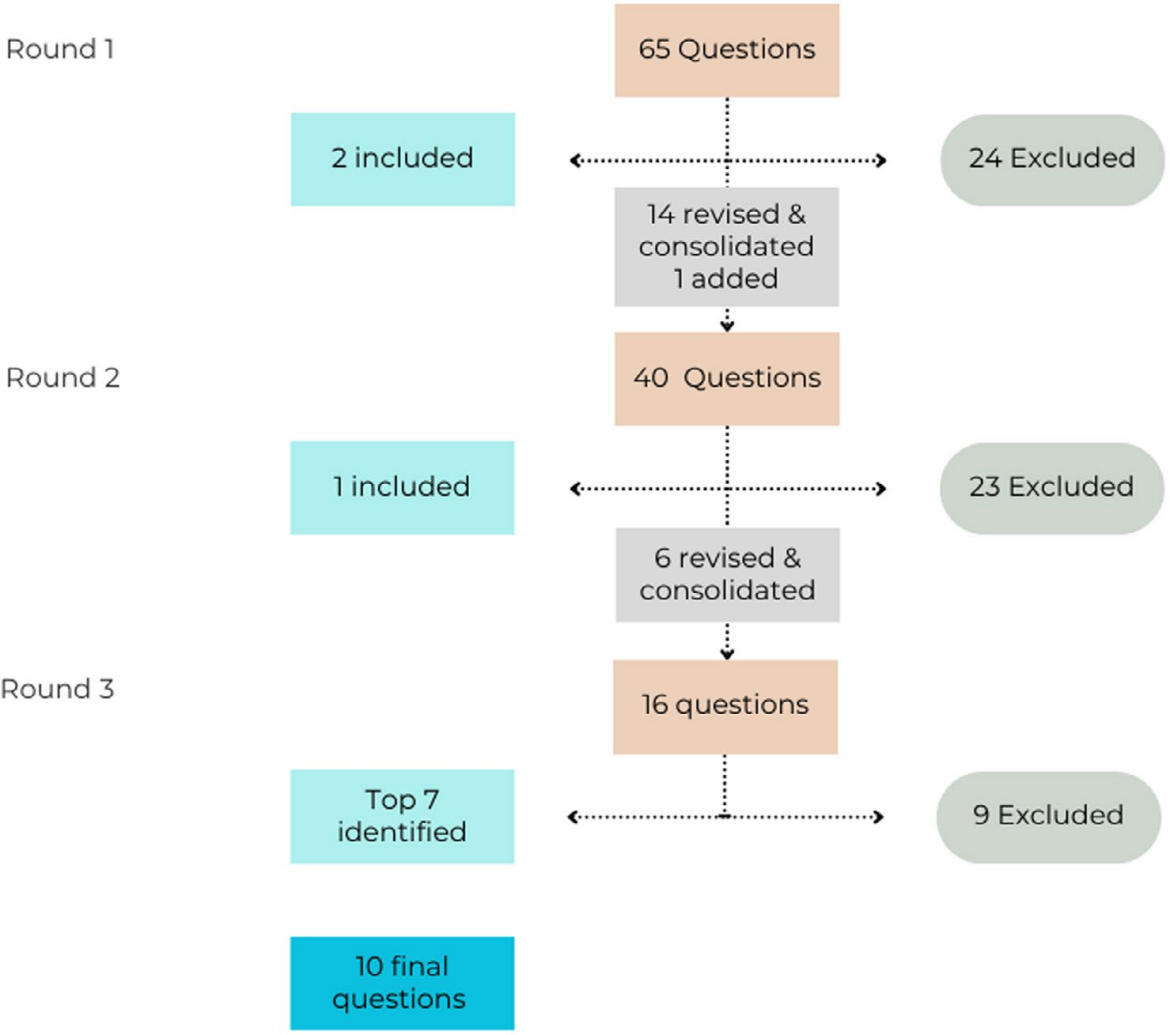
Flow diagram representing question inclusion/exclusion after each delphi round.

Survey response rates were 100%, 92%, and 92% for each Delphi round, respectively. See Figure 1 for a visual depiction of question inclusion and exclusion for each Delphi round. See Data S2 for the full question list and round‐by‐round inclusion data.

In Round 1, a total of 65 questions were available for review. One question met the inclusion threshold (≥ 70% “definitely include”) and was advanced to the final list. Twenty‐four questions were excluded, and 14 were combined or revised for clarity. One new question was added.

In Round 2, a total of 41 questions were available for review. Two questions met the inclusion threshold and were advanced to the final list. Twenty‐three questions were excluded, and 6 questions were revised and consolidated for clarity. No further questions were added.

After Round 2, a total of 3 questions had met the predefined inclusion threshold and were advanced as priority research questions. To identify the remaining 7 and complete the final list of 10, a third round was conducted. In this final round, 16 questions were available for review. Participants were asked to select up to 10 questions they believed should be prioritized. Based on the total number of votes, the 7 highest‐ranked questions were selected and added to the final research agenda, bringing the total to 10 priority questions. Upon review of these 10 questions, four key categories were identified through group discussion to guide future inquiry on coaching and individualized learning: coaching effectiveness and outcomes, coaching program design, faculty preparation and coaching competencies, and learner‐centered factors (Table 1).

**TABLE 1:** Delphi‐informed research agenda for coaching and individualized learning in competency‐based medical education.

*Coaching Effectiveness and Outcomes* Q1. Does coaching facilitate competency achievement in medical education? Q2. Does coaching help with the attainment of competency, or the growth beyond competency to mastery, or both? Q3. What short‐ and long‐term outcomes should be used to assess coaching effectiveness in CBME?
*Coaching Program Design* Q4. What form of coaching is most effective for CBME in GME (program leadership, peer, faculty, non‐faculty, non‐content expert)? Q5. What are the optimal parameters (frequency, timing, location, environment, etc.) for CBME coaching interactions to be effective? Q6. How can coaching support remediation in CBME? Q7. Are structured tools (e.g., individualized learning plans) effective for CBME coaching?
*Faculty Preparation and Coaching Competencies* Q8. How do we train competent coaches for coaching learners in CBME?
*Learner‐Centered Factors* Q9. How do trainees interpret the coaching process? Q10. What effect does being coached have on learners' self‐assessment skills within CBME?

*Note:* The final ten priority research questions identified through a modified Delphi process clustered into four interrelated domains: coaching effectiveness and outcomes, coaching program design, faculty preparation and coaching competencies, and learner‐centered factors. Each domain reflects key areas of inquiry represented within the final priority questions and together frames a focused, practice‐relevant research agenda for coaching within CBME.

## 4 Implications

There remains a critical need to assess the effectiveness of coaching within the CBME framework and how to optimally incorporate it within EM training curricula. The purpose of our process was to identify priority research questions to address key knowledge gaps related to the design, implementation, and outcomes of coaching and individualized learning; scholarly focus on these research questions can assist our specialty in standardizing an evidence‐based approach on how to design and implement effective coaching programs within a CBME framework. This collaborative effort drew upon diverse expertise in CBME, coaching, and medical education scholarship. Through this consensus‐building process, it became evident that coaching within EM's transition to CBME is at a pivotal moment, shifting from broad conceptual endorsement and scattered implementation towards a more rigorous, evidence‐informed understanding of its mechanisms, effectiveness, and best practices.

The final 10 research questions reflect this shift and categorize into four domains of focus, each highlighting a critical area where additional inquiry is needed. These domains—pertaining to coaching effectiveness and outcomes, optimal coaching program design, faculty development, and learner‐centered considerations—represent distinct yet interconnected priorities for advancing the field.

### 4.1 Coaching Effectiveness and Outcomes

A foundational area of inquiry centers on whether coaching achieves its intended outcomes, such as helping learners progress towards competency and, ideally, mastery (Q1 and Q2). This line of questioning is essential to establish before expanding coaching programs or refining delivery models. Identifying and defining short‐ and long‐term markers of coaching effectiveness will be crucial to building an evidence base for its use in CBME (Q3), as well as justification for the investment required to support coaching programs. The inclusion of these questions underscores a shared recognition that measurable, meaningful outcomes are necessary to evaluate the value of coaching interventions. However, assessing coaching effectiveness is inherently complex. It depends on multiple interacting factors, including coach training and fidelity to a coaching model, the quality of the coaching relationship, learner readiness and engagement, and alignment with institutional goals. Learner outcomes may span a range of domains—from improved self‐assessment and goal‐setting to enhanced motivation, mindset, and clinical performance. As a community, we must collectively determine which of these outcomes should be prioritized within a CBME framework and how best to measure them.

### 4.2 Coaching Program Design and Institutionalization

Advancing research on the optimal design and implementation of coaching programs is essential to guide how these interventions can be effectively and feasibly integrated into training environments. This domain of inquiry acknowledges that coaching is not a one‐size‐fits‐all process; its success can depend on multiple contextual factors, including the identity and preparation of the coach (Q4), the timing and setting of coaching interactions (Q5), learner investment in the coaching process, and the presence of tailored support for learners requiring remediation (Q6). Although the resources and infrastructure available across training programs may vary, a clearer evidence base could help identify which coaching models are most effective and scalable, offering a potential standard or set of best practices that others might adapt. This domain also highlights the growing interest in tools that can support coaching, such as ILPs, and calls for a deeper understanding of how such tools can enhance coaching outcomes and learner development (Q7). Given the complexity of this area, a diverse set of methodological approaches will be needed to generate meaningful insights. Both quantitative and qualitative designs, as well as realist evaluation and mixed‐methods studies, may be particularly valuable in capturing the nuanced factors that influence coaching implementation and effectiveness across diverse educational settings.

### 4.3 Faculty Preparation and Coaching Competencies

The identification of core coaching competencies reflects the need to establish shared expectations for the knowledge, skills, and attitudes required for effective practice. As this is a unique approach and skillset separate from the more common educator roles of advisor and mentor, it is crucial that further research identifies how best to train faculty (or other personnel) as coaches (Q8). Furthermore, there remains a need to identify how best to acquire these skills and achieve competencies in terms of training format and frequency (introductory and ongoing development). While there remains a need for rigor in ensuring faculty are well‐prepared for this role, there also remains a need to ensure this skills‐acquisition is accessible and feasible across our specialty in order to standardize approach and delivery. This clarity is necessary to design faculty development initiatives, ensure consistency across programs, and ultimately evaluate coaching quality in CBME.

### 4.4 Learner‐Centered Considerations

The understanding of learner interpretation and perception of the coaching process is critical to successful implementation (Q9). This domain recognizes that learner engagement is shaped by both personal attributes and broader institutional structures, including the quality of the coach–learner relationship and the presence of psychological safety. Each of these factors can impact the effectiveness of the coaching approach and a learner's educational experience. As CBME is learner‐centric, it is imperative that learners' interpretation of the coaching process and their individual needs are central to the design of a broader program. It is also paramount to create a process that hones learners' self‐reflection and self‐assessment skills to ensure they have the tools to continue to grow and learn successfully after training (Q10). Delineating specific features of coaching that lead to learner growth and development within the context of CBME, and how best to engage and support each individual learner in this framework, will support broad implementation of effective coaching programs across GME.

These selected questions and categories focus research on the influence of both individual and contextual factors and acknowledge the complexity of implementing coaching in real‐world settings. These priorities suggest a field ready to operationalize coaching as a rigorously defined, measurable, and contextually adaptable educational strategy to best serve its learners in a CBME framework.

### 4.5 Comparison to Prior Work

This work aligns with prior work in CBME, which identifies coaching as an essential strategy for supporting learner development [18, 36]. Similarly, it emphasizes the understanding of coaching contexts and the quality of coach‐learner relationships that are most effective in driving learner engagement. Additionally, it outlines the nuanced challenges that exist in implementing CBME broadly [36].

This work diverges from the current literature base in that it represents the first specialty‐specific, consensus‐driven research agenda for coaching in GME. Coaching frameworks have been proposed in other contexts [37, 38], but a discipline‐specific approach for EM provides a model for our specialty to consider how to best integrate coaching into our own training programs to meet the specific needs of our EM learners. Given the numerous clinical environments that our trainees practice in, the inconsistency of clinical team personnel on a shift‐by‐shift basis, and variability of clinical exposure, there are unique considerations that we need to take into account for our EM learners when developing strategies to implement coaching and individualized learning in the broader CBME format. In addition, posing questions to target measurable coaching outcomes that go beyond anecdotal satisfaction enables evidence‐based integration into educational programs. Offering a targeted set of research priorities can shape scholarship and practice across the broader GME landscape.

### 4.6 Future Implications

The potential impact of this work spans multiple stakeholders, including researchers, GME leadership, and accreditation bodies. For researchers, these findings offer a roadmap for the design and implementation of high‐impact studies. By aligning scholarship priorities, there is an opportunity to foster multi‐institutional, longitudinal investigation into the coaching implementation and impact. Defining short‐ and long‐term outcomes provides the necessary foundation for developing and validating tools that measure coaching impact, further enhancing the evidence base for coaching in CBME and supporting integration into GME programs.

For GME program leadership and administration, the results underscore the importance of deliberate coaching integration in CBME. Aligning individualized learning plans, coaching context and interactions, and competency assessments with measurable outcomes can enhance program effectiveness. Furthermore, understanding the circumstance under which coaching is most effective allows faculty to tailor approaches to learners' specific needs, supporting equity in training.

For accrediting bodies, this work offers an evidence base to inform standards and expectations for coaching integration in GME. Although developed in the context of emergency medicine, the outcomes have the potential to guide faculty development and specialty‐specific standards for coaching across GME programs.

### 4.7 Challenges and Considerations

Several limitations of this work should be acknowledged. The expert consensus group was drawn primarily from academic EM, and while the process assured diverse representation, the results may require adaptation to other specialties if desired. Additionally, this work was conducted within a single expert workgroup; future studies should evaluate and validate these priorities with external stakeholders, including the resident/fellow learners aimed to serve. The Delphi process achieved consensus, but may have excluded novel or emerging ideas. The group discussion may have inadvertently overshadowed opinions of the minority in an effort to achieve consensus; however, individuals were given the opportunity to independently vote and offer free‐text commentary in between Delphi rounds in an effort to consider all opinions. Additionally, the represented workgroup members all had some expertise with coaching and individualized learning, thus potentially overlooking a broader range of perspectives from other stakeholders that were not invited to participate. The literature review was limited to the past 10 years as an arbitrary cut off to balance recency and relevance with practicality.

The selected ten final questions highlight the most critical priorities identified by the expert consensus group. However, there remain several other important factors—such as institutional factors (current policies, accessibility to resources, funding opportunities), types of coaching (coaching in‐the‐moment, longitudinal coaching, wellbeing coaching), learner factors (lived experiences, professional identity, cultural diversity), the emerging use of artificial intelligence (AI) and its implications on coaching, and the broader impact of coaching on not only individual trainees, but general patient‐ and population‐health—that continue to have a need for further investigation as well. The pragmatic need to focus the research agenda rendered a limitation on including other relevant and necessary questions.

Implementation of this work will also be challenging. Variability in coaching expertise, competing clinical demands, and institutional support may limit the scalability of coaching in GME. Understanding coaching outcomes is a priority, but integration must allow for flexibility that addresses local contexts and resident needs. Similarly, the sustainability of GME coaching programs will be dependent upon institutional commitment to protected time, evaluation of impact, and iteration as evidence‐based understanding of coaching programs grows.

Despite these challenges and limitations, the identified priorities represent an important step in operationalizing coaching as a core piece of CBME in EM. Continued collaboration amongst researchers, GME leadership, and accreditation administration can guide the development of evidence‐based coaching practices that support individual learner growth and, ultimately, patient care outcomes.

## Author Contributions

Study concept and design (All), acquisition of data (All), analysis and interpretation of the data (All), drafting of the manuscript (All), critical revision of the manuscript for important intellectual content (All), statistical expertise (n/a), obtained funding (HCW), administrative, technical, or material support (N.Z.), study supervision (HCW).

## Funding

The 2025 SAEM Consensus Conference was funded by the AMA Reimagining Residency Grant, PI—Holly Caretta‐Weyer.

## Disclosure

Artificial intelligence was not used in the preparation of this manuscript.

## Conflicts of Interest

Sally A. Santen: AMA consultant. Michele L. Dorfsman: Owner of Physician Coach MD LLC. Holly Caretta‐Weyer: AMA Reimagining Residency Grant funding. All other authors declare no conflicts of interest.

## Supporting information


**Data S1:** aet270148‐sup‐0001‐Supinfo1.docx.

**Data S2:** aet270148‐sup‐0002‐Supinfo2.docx.

## Data Availability

The data that support the findings of this study are available on request from the corresponding author. The data are not publicly available due to privacy or ethical restrictions.
