# Supplementary material for: Coaching and Individualized Learning in Competency‐Based Medical Education: Framework Development and Research Priorities
Source: AEM Educ Train. 2026 Apr 14;10(2):e70148. doi: 10.1002/aet2.70148 (PMC13079412; doi:10.1002/aet2.70148)
Supplement: Supplementary file 1 — Data S1: aet270148‐sup‐0001‐Supinfo1.docx. [file AET2-10-e70148-s001.docx]

**Supplement 1: Participants for the 2025 SAEM Consensus Conference Workgroup for “Coaching and Individualized Learning”**

| **Author Name and Title** | **Institution and Department** | **Experience in Coaching, Individualized Learning, and**  **Graduate Medical Education** |
| --- | --- | --- |
| **Simanjit K. Mand, MD**  Assistant Professor | University of Wisconsin-Madison School of Medicine and Public Health  Department of Emergency Medicine | Director of University of Wisconsin EM Residency Coaching Program  Publications on coaching in GME  Physician Coaching Institute training with Level 1 Certification  Assistant Fellowship Director for Advanced Emergency Medicine Ultrasonography    Previous Assistant Residency Program Director |
| **Meg Wolff, MD** Professor | University of Michigan Medical School  Department of Emergency Medicine & Pediatrics | Director of Coaching Program for University of Michigan Medical School  Publications on coaching, including author of AMA handbooks, editor of coaching text  Coach for medical students, residents, fellows, and faculty  Previous Associate Residency Program Director  Previous Associate PEM Fellowship Program Director |
| **Sarah R. Williams, MD, MHPE, PCC**  Clinical Professor | Stanford University School of Medicine  Department of Emergency Medicine | Director of Stanford EM Residency Coaching Program  Founding Executive Director of COACHME at Stanford (accredited national coach training program for medical educators seeking board certification in coaching)  Publications on coaching  PCC (ICF) and BCC Professional Certifications in Coaching  Professional coach for medical students, residents, fellows, and faculty  Director of Stanford Health Professions Education and Scholarship Program (medical education certificate program)  Previous EM Residency Director, APD, and Ultrasound Fellowship Director |
| **Sally A. Santen, MD, PhD**  Professor | University of Cincinnati College of Medicine  Department of Emergency Medicine and Medical Education | Associate Dean of Medical Education Research and Innovation  PhD in Education  Scholar in coaching  Faculty coach |
| **Kevin R. Scott, MD MSEd**  Associate Professor | Geisinger Commonwealth School of Medicine  Department of Emergency Medicine | Former Founding Director of the EM Individualized Learning and Coaching Program at University of Pennsylvania  Publications on coaching in GME |
| **Alexander Garrett, MD**  Acting Assistant Professor | University of Washington School of Medicine  Department of Emergency Medicine | Fellowship training in Medical Education Research |
| **Charles Brown, MD**  Assistant Professor | Oregon Health & Science University School of Medicine  Department of Emergency Medicine | Director of Assessment and Feedback for OSHU EM Residency |
| **Jung G. Kim, PhD, MPH**  Assistant Professor | New York University Grossman School of Medicine  Department of Emergency Medicine | PI for NIH-funded study on ACGME milestones  Advanced Practitioner Certification Team Diagnostic Survey |
| **Michele L. Dorfsman, MD**  Professor | University of Pittsburgh School of Medicine  Department of Emergency Medicine | Longitudinal coach for medical students at UPSOM, Clinical Educator Faculty  Publications on coaching  Professional coaching business  Independent contractor |
| **Nicole Deiorio, MD**  Professor | Virginia Commonwealth University School of Medicine  Department of Emergency Medicine | Director of two coaching programs  Educational research on coaching and CBME  Certified coach |
| **Jeremy Branzetti, MD, MHPE**  Associate Professor | Yale University School of Medicine  Department of Emergency Medicine | ACC (ICF) Professional Certification in Coaching  Owner of professional and developmental coaching business |
| **Daniel P. Runde, MD, MME**  Clinical Professor | University of Iowa College of Medicine  Department of Emergency Medicine | Fellowship in Medical Education |
