# Supplementary material for: Coaching and Individualized Learning in Competency‐Based Medical Education: Framework Development and Research Priorities
Source: AEM Educ Train. 2026 Apr 14;10(2):e70148. doi: 10.1002/aet2.70148 (PMC13079412; doi:10.1002/aet2.70148)
Supplement: Supplementary file 2 — Data S2: aet270148‐sup‐0002‐Supinfo2.docx. [file AET2-10-e70148-s002.docx]

**Supplement 2: Proposed Research Questions and Delphi Round-by-Round Data**

**Proposed Research Questions**

1. Does coaching facilitate competency attainment in medical education?
   1. How does coaching facilitate competency attainment (can we test a theory of change)
2. Does coaching have to be faculty or can it be peers?
   1. Related/broader: who is best positioned to serve as coach? (GME leadership, faculty, peers, external?)
   2. The general literature supports Non-content domain experts as coaches. So maybe the question is are there areas in medical education where a peer coach or content expert coach is needed?
3. How do we train people to coach?
   1. What is minimum amount of training necessary?
4. Do you have to have direct observation to effectively coach for CBME?
   1. This is sub-question of what is coaching in medical education- is it performance /feedback based coaching or is it ICF-defined?
5. How do we measure/”prove” coaching has occurred?
6. How do you engage intrinsic motivation into CBME? Make it fulfilling beyond a “checklist” exercise
7. How to best create psychologically safe spaces (and promote trust) for coaching in CBME?
8. What do trainees require at minimum in terms of coaching?
   1. Frequency of meetings, type of coaching to best support CBME
9. Are there designated coaches or does it apply to all faculty members?
10. Is there a preferred coach training program/initiative?
11. What outcomes are important?
12. How does coaching differ in cases of those trainees on remediation versus those that are not identified as such?
13. “Where” does coaching occur?
14. Is coaching in CBME limited to performance based coaching?
15. Can we”/do we meaningfully differentiate between coaching and mentorship?
16. How do we reconcile the external assessment of CBME/CBA with the internally generated definition of success favored by coaching?
17. Does coaching help with attainment of competency, or the growth beyond competency to mastery (or both)? G
18. Is coaching a meaningful intervention for learners who struggle to achieve competency (or less gentle, “incompetent learners”).
19. How do trainees interpret the coaching process?
20. Is it reasonable for trainees to coach each other (senior resident to junior resident)?
21. Is there an optimal time for coaching to begin?
22. In what ways can interpersonal and systemic biases influence the coaching relationship?
23. The role of identity safety in facilitating healthy coaching relationships.
24. How to interpret coaching in CBME through existing theoretical lenses (educational alliance, self-regulated learning, co-regulated learning)
25. Explore the goal-setting process in coaching: how do goals evolve over time/as trainees progress along milestones?
26. How is trust built in the coaching relationship?
27. Is coaching associated with faster attainment of competency
28. What is the optimal frequency / format for coaching interactions to be effective?
29. Who is an ideal coach? (separating evaluation from coaching? Faculty who are invested in learners? Learner-elected and selected? etc.)
30. How do we best support our coaches in their role? (training, buy-down, CME for development)
31. Does coaching during training actually help develop the master adaptive learner? (how do we assess this?
    1. How do we measure the long-term outcomes of coaching (beyond training?)? What do we measure?
32. How can we prove/measure return on investment for coaching related to CBME? What’s in it for the stakeholders? Who are the stakeholders? (Departments, institutions, society, medical boards, professional liability considerations, etc…)
33. How best to normalize the coaching approach to learners and faculty who have been in “fixed mindset” hierarchies for most of their professional lives?
34. Coaching for competency or coaching for self-regulated learning (i.e. ability for informed self-assessment and future planning of learning)? Is there a “better” way to attain competency?
35. Are individualized learning plans or other similar tools necessary for effective coaching?
36. Is our ability to assess CBME rigorous enough to be able to accurately gauge/attribute the impact of coaching in this arena?
37. Video review of learner performance with a coach: any additional benefit over/instead of real-time coaching or feedback?
38. “Warm-up” coaching immediately before an experience–I feel this is underexplored in general and a lot of potential questions here
39. Are there differences that predict length of individual coaching sessions needed between similar groups?
40. Additional data to support coaching’s effects on different wellbeing metrics
41. Are there any negative effects of coaching, for example as discerned by a qualitative study of coachees?
42. How can true coaching (not skills coaching) help with remediation for professionalism/communication deficits?
43. Does EPA coaching affect the speed at which learners become entrustable?
44. Does being coached eventually improve learners’ own self-assessment skills?
45. Non-inferiority of peer coaching as compared to faculty coaches?
46. Is an individual coach “assigned” throughout training more/less effective than having a more generalizable approach to coaching?  If so, how are coaches/coachees matched?
    1. If coaching to multiple domains (skills/technical-based, professional development, well-being, etc.), is it more effective to have a single coach for all domains or a separate coach for each domain?
47. How may one’s image/professional identity be affected or challenged by the coaching apparatus? How do culture, image, and professional identity intersect in these relationships?
48. Does adding mandatory coaching add to burden on coaches and trainees? How to alleviate impression of increased time demands from this requirement?
49. Meta: how do we coach the coaches? Peer-coaching for faculty development?
50. How much “coaching” in GME is actually coaching?
51. What are the impacts of faculty-to--faculty coaching programs on implementation of CBME?
52. Do faculty-to-faculty coaching programs work? What are the impacts?
53. How much coaching is needed for a particular impact/effect?
54. What are the barriers/facilitators of coaching within the context of cbme?
55. Utility coach: can single coach serve multiple coaching roles to address individual learners needs?
56. Are there benefits to coaching targeted toward personality traits? (inflexibility, laziness, etc.)
57. What differentiates coaching from mentorship, advising, or feedback?
58. Is coaching more impactful for hard skill formation (procedures) than for soft skill formation (task switching, efficiency, gestalt)?
59. It is well established that learners receive feedback when it comes from a trusted source, does this apply to coaching as well? How can we help learners trust coaches?
60. What do learners hope to gain from the learner-coaching relationship?
61. Is coaching more effective in person versus virtually?
62. Does coaching in team training simulation help outcomes. Article:There is very low evidence that conditions (such as coaching) effect the transfer of learning/ outcomes after  team simulation training. (wittig)
63. Surgery has a model of expertise coaching (e.g. New Yorker- Atul Gwande) would the model be acceptable in EM. Are there coaching programs using similar model in EM? Is it effective? Acceptable? scalable ? (article about surg expertise -Sachdeva
64. Does coaching (by physician) of non technical skills improve… (surgery study, Yule
65. Use of AI coaching (china based study of AI coaching laparoscopic skills RCT . wu)
66. Assessment of goal setting quality with coaching
67. Assessment of coaching quality (surg study postoperative debriefs between coach/coachee pairs, co-coding themes based on established principles of effective coaching: (i) self-identified goals, (ii) collaborative analysis, (iii) constructive feedback, and (iv) action planning. praderelli)
68. The influence of clinical coaching teams on quality of entrustable professional activity assessments (singh)
69. How do we incorporate peer teaching/coaches into EM residency?
    1. How does this apply to the EM proposal for 4 year programs?
    2. And should coaching be an ACGME core competency for residents?
70. What is the evidence that coaching enhances skills acquisition in clinical environments? (Wittig et al); ROI
71. Two great examples of the above: short video debriefs of surgical skills with coaching had great outcomes compared to controls (Soucisse et al; Tanaka et al)
72. Coaching and its role in adaptive expertise over the arc of a career (surgical/procedural) (Sachdeva et al; Williams et al (TEAMS)
73. What are ways to measure the impact of procedural skills coaching beyond improvement of the skill itself (physiologic, HR variability, etc–Timberlake suturing study example, HR variability suggesting better coping–implications on well being?)
74. How may we use coaching to support feedback conversations? Peer coaching acceptable in surgical culture); Praderelli et al as an example
75. What is the role of AI in technical skills coaching (good outcomes and acceptable in Wu study); implications?
76. What may be the role(s) of AI in coaching in general? Implications, ethics, integration with authentic human communication and connection?
77. Recommendations of coaching by specialty societies–how many have done so? (Sachdeva et al)
78. What is the role of coaching and deliberate practice in nontechnical domains? (ie improved communication) (Rivet et al)
79. Role of coaching in assessment?
    1. (Vallevand et al); divergence around assessment of professionalism construct;
    2. What are the implications on the quality of the assessments (EPA as example: Singh); higher quality feedback found in other studies–implications?
80. What are examples of ways to develop coaching as a skillset (for trainees and faculty): Role of CBME? Curricula? (van Vendeloo et al; Zhu et al; Siriani et al; Shiozawa et al)
81. Potential impact of coaching on patient safety? (Yule et al: called for help sooner in simulated bleeding scenarios); ROI (also relates to clinical skills transfer above, Tanaka, Soucisse examples)
82. Making a case: ROI of coaching (clinical outcomes, patient safety, skills acquisition during residency and maintenance of skills over career…)
83. What are good examples of reflection exercises with a coach (portfolios, etc.), and how to preserve safe space/due process (Schuwirth, Van Der Vleuten)
84. How to best integrate coaching into leadership development? (Sadowski et al)
85. How to best support learners with structured goal-setting opportunities? Implications for programs? (SMARTer, Winkel et al)
86. Professional identity formation: coaching’s role? (Toh et al scoping review of the “mentorship umbrella”)
87. Coached to Coach: how do we train coaches?
88. Faculty development programs: most efficient and effective, appealing to faculty. Best practices?
89. What is the actual definition of coaching in CBME?
90. Is the development of competency/milestones champions a good idea in developing a training program for coaches? How is this different than simply training the coaches?
91. Is it helpful to train faculty in a set of EPAs to develop their mindset when adopting a CBME model?
92. What are the barriers to development of high quality EPA assessments in a training program?
93. What is the role of high fidelity simulation in CBME?
94. Which is more effective–observation of a holistic skill set in a simulation environment, or a coaching model with interactive coaching as part of the simulation. Or are both necessary?
95. In a pediatric curriculum, is a longitudinal approach more effective due to spaced repetition versus scheduled block rotations?
96. Note: could not open “Necessary but Not Sufficient: Identifying Conditions for Effective Feedback during Internal Medicine Residents' Clinical Education”
97. What are the benefits of simulated teaching in coaching the coaches (faculty)?
98. What is the role of peer coaching in CBME? Meaning evaluation by co-trainees.
99. What is the role of self assessment in CBME?
100. Could we develop remote systems of coaching for multiple institutions to maximize training? Either in recorded videos or contemporaneously via a video platform.
101. Should institutions be developing simulation training/assessments to help faculty maintain skills throughout their career?

**Delphi Round 1 Questions**

1. **Does coaching facilitate competency attainment in medical education?**

******advanced to final round after voting in Delphi Round 1******

1. How do we interpret coaching in CBME through existing theoretical lenses (educational alliance, self-regulated learning, co-regulated learning)?
2. Does coaching help with the attainment of competency, or the growth beyond competency to mastery (or both)?
3. What is the best way to actually coach for competency: Aim to coach for competency itself, or coach for self-regulated learning (i.e., ability for informed self-assessment and future planning of learning)?
4. How do the differences between coaching and mentorship and advising affect competency development?
5. Who is best positioned to serve as coach within CBME? (GME leadership, faculty, peers, external?)
6. Are faculty using coach-like skills as effectively as dedicated coaches?
7. What is the evidence of peer coaching as compared to faculty coaches?
8. How should coaches be selected in CBME? Core faculty? Faculty who are invested in learners? Learner-elected and selected? etc.
9. What is the ideal type or level of training needed to coach within CBME?
10. What is the minimum amount of training necessary to coach within CBME?
11. Is there a preferred coach training program/initiative?
12. How do we best support our coaches in their role? (training, buy-down, CME for development)?
13. Meta: how do we coach the coaches? Peer-coaching for faculty development?
14. How should coaching effectiveness be assessed in CBME
15. What are the competencies for coaches within CBME?
16. What is the optimal frequency/format for CBME coaching interactions to be effective?
17. Where should coaching within CBME occur (ie clinical learning environment? Outside of CLE?)
18. What is the role of longitudinal relationship in quality of coaching for CBME?
19. If coaching to multiple domains (skills/technical-based, professional development, well-being, etc.), is it more effective to have a single coach for all domains or a separate coach for each domain?
20. Are structured tools (e.g., individualized learning plans or other similar tools) necessary for effective coaching?
21. How do we measure/“prove” coaching has occurred?
22. Is coaching associated with faster attainment of competency?
23. Does coaching during training actually help develop the master adaptive learner? (how do we assess this?)
24. How do we measure the long-term outcomes of coaching (beyond training)? What do we measure?
25. What is the return on investment for coaching related to CBME? What’s in it for the stakeholders? Who are the stakeholders? (Departments, institutions, society, medical boards, professional liability considerations, etc.?)
26. Is our ability to assess CBME rigorous enough to be able to accurately gauge/attribute the impact of coaching in this arena?
27. What data or input do we need to be able to accurately assess the impact of coaching in CBME?
28. What additional data is needed to assess coaching’s effects on different well-being metrics (including retention, workforce considerations)
29. What do trainees require at minimum in terms of coaching?
30. What preparation do trainees require to successfully engage with coaching in CBME?
31. How do trainees interpret the coaching process?
32. What effect does being coached have on  learners’ self-assessment skills within CBME?
33. How may one’s image/professional identity be affected or challenged by the coaching apparatus?
34. How do culture, image, and professional identity intersect in coaching relationships within CBME?
35. How best to normalize the coaching approach to learners and faculty who have been in “fixed mindset” hierarchies for most of their professional lives?
36. What is the role of identity safety in facilitating healthy coaching relationships in CBME
37. What has a bigger impact on competency? A coach (ie person), or coaching culture (ie group growth mindset)?
38. How to best create psychologically safe spaces (and promote trust) for coaching in CBME?
39. Do you have to have direct observation to effectively coach for CBME?
40. What is the role of video review of learner performance with a coach? Any additional benefit over/instead of real-time coaching or feedback?
41. What is the role of “warm-up” coaching immediately before a learning experience?
42. How do goals evolve over time/as trainees progress along milestones?
43. What [coaching? Learner? Etc.] outcomes are important?
44. How to engage intrinsic motivation into CBME? Make it fulfilling beyond a “checklist” exercise
45. How does coaching differ in cases of those trainees on remediation versus those that are not identified as such?
46. Should coaching differ for those on remediation versus those that are not?
47. Is coaching a meaningful intervention for learners who struggle to achieve competency (or less gentle, “incompetent learners”)?
48. How can feedback content and culture be enhanced through coaching in CBME?
49. How to optimize “acceptability” of coaching within medical workflows/culture (great literature in the surgical literature about short debriefings impacting surgical skill outcomes)
50. Does EPA coaching affect the speed at which learners become entrustable?
51. How do we reconcile the external assessment of CBME/CBA with the internally generated definition of success favored by coaching?
52. Is coaching in CBME limited to performance-based coaching?
53. Does coaching affect the speed at which learners attain competency?
54. How do we maintain the positive learning climate and avoid the pitfalls of over-assessment exhaustion (this relates to CBME in general and maybe coaching can help mitigate this/help provide a different lens through which to view it?)
55. What is the role of coach in the competency committee?
56. What is the role of the competency committee in feeding into/driving coaching
57. How can true coaching (not skills coaching) help with remediation for professionalism/communication deficits?
58. Are there differences that predict the length of individual coaching sessions needed between similar groups?
59. Are there any negative effects of coaching - on learners, faculty, institutions, etc.?
60. Does adding mandatory coaching add to burden on coaches and trainees?
61. Does mandatory coaching work?
62. Should coaching in CBME be voluntary or mandatory?
63. How to alleviate the impression of increased time demands (on coaches and learners) of coaching within CBME?
64. What is the role of AI?

**Delphi Round 2 Questions**

1. What theoretical concepts underpinning coaching (e.g. educational alliance, self-regulated learning, co-regulated learning, positive psychology, psychological safety, etc.) impact competency development?
2. Does coaching help with the attainment of competency, or the growth beyond competency to mastery (or both)?
3. Which is more impactful on competency achievement - coaching for competency or coaching for self-regulation of learning?
4. How do the differences between coaching and mentorship and advising affect competency development?
5. **What form of coaching is most effective for CBME in GME (program leadership, peer, faculty, non-faculty, non-content expert)?**

******advanced to final round after voting in Delphi Round 2******

1. What training is needed to coach for CBME in GME?
2. How do we train the coaches?
3. How do we best support our coaches in their role? (training, buy-down, CME for development)?
4. **What short- and longer-term outcomes should be used to assess coaching effectiveness in CBME?**

******advanced to final round after voting in Delphi Round 2******

1. What are the competencies for coaches within CBME?
2. What is the optimal frequency for CBME coaching interactions to be effective?
3. What is the optimal environment (timing, location, proximity to clinical learning, etc.) for coaching to occur?
4. What is the role of longitudinal relationship in quality of coaching for CBME?
5. If coaching to multiple domains (skills/technical-based, professional development, well-being, etc.), is it more effective to have a single coach for all domains or a separate coach for each domain?
6. Are structured tools (e.g., individualized learning plans or other similar tools) necessary for effective coaching?
7. How do we measure/“prove” coaching has occurred?
8. Is coaching associated with faster attainment of competency?
9. How do we measure the long-term outcomes of coaching (beyond training)? What do we measure?
10. What is the return on investment for coaching related to CBME?
11. Who are the stakeholders for coaching in CBME (departments, institutions, society, medical boards, professional liability considerations, etc.)?
12. What preparation do trainees require to successfully engage with coaching in CBME?
13. How do trainees interpret the coaching process?
14. What effect does being coached have on learners' self-assessment skills within CBME?
15. How do culture, image, and professional identity intersect in coaching relationships within CBME?
16. How best to normalize the coaching approach to learners and faculty who have been in "fixed mindset" hierarchies for most of their professional lives?
17. What is the role of identity safety in facilitating healthy coaching relationships in CBME?
18. How to best create psychologically safe spaces (and promote trust) for coaching in CBME?
19. What is the role of video review of learner performance with a coach in CBME?
20. How do goals evolve over time/as trainees progress along milestones?
21. What [coaching? Learner? Etc.] outcomes are important?
22. How does coaching differ for trainees on remediation versus those who are not identified as such?
23. Is coaching a meaningful intervention for learners who struggle to achieve competency?
24. How can feedback content and culture be enhanced through coaching in CBME?
25. How do we reconcile the external assessment of CBME/CBA with the internally generated definition of success favored by coaching?
26. What is the role of coach in the competency committee?
27. What is the role of the competency committee in feeding into/driving coaching?
28. How can true coaching (not skills coaching) help with remediation for professionalism/communication deficits?
29. Are there any negative effects of coaching - on learners, faculty, institutions, etc.?
30. Does mandatory coaching work?
31. Should coaching in CBME be voluntary or mandatory?
32. What is the role of AI in coaching for CBME?

**Delphi Round 3 Questions**

1. Does coaching help with the attainment of competency, or the growth beyond competency to mastery (or both)?
2. What theoretical concepts underpinning coaching (e.g., education alliance, self-regulated learning, co-regulated learning, positive psychology, psychological safety, etc.) impact competency development?
3. How do we train competent coaches for coaching learners in CBME?
4. What are the competencies (such as psychologically safe spaces, promoting trust, etc.) for coaches within CBME?
5. What are the optimal parameters (frequency, timing, location, environment, etc.) for CBME coaching interactions to be effective?
6. What is the role of longitudinal relationship in quality of coaching for CBME?
7. If coaching to multiple domains (skills/technical-based, professional development, well-being, etc.), is it more effective to have a single coach for all domains or a separate coach for each domain?
8. Are structured tools (e.g., individualized learning plans or other similar tools) necessary for effective coaching?
9. What preparation do trainees require to successfully engage with coaching in CBME?
10. How do trainees interpret the coaching process?
11. What effect does being coached have on learners' self-assessment skills within CBME?
12. How does medical culture, learning environment, image, and professional identity influence coaching relationships within CBME?
13. How do goals evolve over time/as trainees progress along milestones?
14. How can coaching support remediation in CBME?
15. Are there any negative effects of coaching - on learners, faculty, institutions, etc.?
16. Should coaching in CBME be voluntary or mandatory?
